# Chronic Limb Remote Ischemic Conditioning may have an Antihypertensive Effect in Patients with Hypertension

**DOI:** 10.14336/AD.2021.0604

**Published:** 2021-12-01

**Authors:** Wenting Guo, Changhong Ren, Bowei Zhang, Wenbo Zhao, Yu Gao, Wantong Yu, Xunming Ji

**Affiliations:** ^1^Department of Neurology, Xuanwu Hospital, Capital Medical University, Beijing, China.; ^2^Beijing Key Laboratory of Hypoxia Conditioning Translational Medicine, Xuanwu Hospital, Capital Medical, Beijing, China.; ^3^Beijing Municipal Geriatric Medical Research Center, Beijing, China.; ^4^Department of Neurosurgery, Xuanwu Hospital, Capital Medical University, Beijing, China.; ^5^Department of Neurology, The Affiliated Hospital of Qingdao University, Qingdao, Shandong, China.

**Keywords:** remote ischemic conditioning, hypertension, antihypertensive effect, blood pressure

## Abstract

Hypertension is the leading preventable risk factor for all-cause morbidity and mortality worldwide. Despite antihypertensive medications have been available for decades, a big challenge we are facing is to increase the blood pressure (BP) control rate among the population. Therefore, it is necessary to search for new antihypertensive means to reduce the burden of disease caused by hypertension. Limb remote ischemic conditioning (LRIC) can trigger endogenous protective effects through transient and repeated ischemia on the limb to protect specific organs and tissues including the brain, heart, and kidney. The mechanisms of LRIC involve the regulation of the autonomic nervous system, releasing humoral factors, improvement of vascular endothelial function, and modulation of immune/inflammatory responses. These underlying mechanisms of LRIC may restrain the pathogenesis of hypertension through multiple pathways theoretically, leading to a potential decline in BP. Several existing studies have explored the impact of LRIC on BP, however, controversial findings were reported. To explore the potential antihypertensive effect of LRIC and the underlying mechanisms, we systematically reviewed the relevant articles to provide an insight into the novel therapy of hypertension.

## 1. Introduction

Hypertension is the leading cause of attributable deaths and burden of disease globally, which is also one of the important preventable risk factors for cardiovascular disease (CVD)[1]. The pathogenesis responsible for hypertension include overactivation of the sympathetic nervous system (SNS) and renin-angiotensin-aldosterone system (RAAS), increased chronic inflammatory responses, and dysregulation of the immune system [2]. Despite great efforts have been made and extensive studies have been conducted over the past decades, the control of hypertension is still not satisfying [3, 4]. This situation requires us to search for novel pharmaceutical or non-pharmaceutical means to optimize the therapeutic approach for hypertension. Limb remote ischemic conditioning (LRIC) may be a new method worthy to be further explored.

LRIC includes repetitive inflation-deflation of a pneumatic cuff on the limb, which can trigger endogenous protective effect to remote organs such as the heart, brain, and kidney by neural, humoral, and immune/ inflammatory pathways [5, 6]. Numerous studies have demonstrated that LRIC is a promising treatment in various clinical conditions, especially in ischemia-reperfusion injuries [5, 7-10]. Studies have explored the impact of LRIC on blood pressure (BP) with controversial results in recent years. The BP-lowering effect of LRIC was observed in several studies [11-17], but the results were not consistent in others [18-24]. Due to the heterogeneity in participant selection and LRIC protocols, it is necessary to make a systematic analysis of these studies to explore whether LRIC has the potential to be a novel therapeutic method for hypertension.

We systematically analyzed the clinical research data about the impact of LRIC on BP which was published on PubMed, EMBASE, Cochrane Library databases, and Web of Science up to Jan 2021, to explore whether LRIC has a BP-lowering effect and its potential mechanisms. In addition, we will provide an overview of the ongoing clinical trials and discuss the challenges we are going to face in the future.

## 2. Whether LRIC has an antihypertensive effect in humans?

22 clinical studies involving 665 participants showed the effect of LRIC on BP according to a comprehensive literature retrieval (see Table 1-2), however, these studies were with heterogeneities. First, different ranges of subjects were included. Both healthy volunteers and CVD patients were enrolled in 2 studies [25, 26], healthy volunteers with normal BP were included in 6 studies [18, 20-22, 24, 27], patients with hypertension or CVD were enrolled in 14 studies [11-16, 19, 23, 28-33]. Second, different LRIC schemes were used. LRIC therapy for once or twice only was performed in 10 studies [21-23, 25-27, 30-33], a long-term repeated LRIC therapy was applied in 12 studies [11-16, 18-20, 24, 28, 29]. Thus, we classified these relevant articles according to different participant selection and LRIC schemes to clarify whether LRIC has an antihypertensive effect in specific populations, the details of these studies are listed in Table 1 and 2.

**Table 1:** The effect of LRIC on BP in healthy volunteers.

Study	No. Participants	Design	Comparator	LRIC protocol	Frequency, Duration	Antihypertensive effect
Kimura [24]	20 healthy	RCT	Control	1×5 min ischemia	6×daily, 1 month	NO
Jones [18]	18 healthy	RCT	Control	4×5 min ischemia/5min reperfusion	3×weekly, 8 weeks	NO
Banks [20]	10 healthy	Cohort	None	4×5 min ischemia/5min reperfusion	1×daily, 9 days	NO
Zagidulin [25]	20 healthy	RCT	Sham control	3×5 min ischemia/5min reperfusion	Once-only	NO
Khaliulin [21]	40 healthy	RCT	Sham control	3×5 min ischemia/5min reperfusion	Once-only	NO
Muller [22]	40 healthy	RCT	Sham control	3×5 min ischemia/5min reperfusion	Once-only	NO
Li [26]	24 healthy	Cohort	None	5×5 min ischemia/5min reperfusion	Once-only	YES
Graua [27]	20 healthy	Cohort	None	4×5 min ischemia/5min reperfusion	Once-only	YES

The cuff pressure is about 200-220mmHg or at 20/50mmHg above systolic BP to induce ischemia.

### 2.1 The effect of LRIC on BP in healthy volunteers

#### 2.1.1 The effect of once-only LRIC on BP

Muller *et al.* carried out a randomized controlled trial (RCT) of 40 young healthy volunteers and found that once-only LRIC treatment consisting of three cycles of 5min ischemia followed by 5min reperfusion did not affect BP [22]. With the same LRIC plan, the same conclusion was drawn from the RCT conducted in healthy subjects by Khaliulin *et al.* and Zagidullin *et al.* [21, 25]. However, other prospective cohorts showed that once-only LRIC therapy could temporarily lead to a drop in BP for healthy people [26, 27]. The above-mentioned results seem insufficient for us to draw a certain conclusion. Therefore, we will further discuss the effect of long-term repeated LRIC on BP in this review (See Table 1).

#### 2.1.2 The effect of repeated LRIC on BP

Kimura *et al.* found that a LRIC scheme which was performed a single 5-min ischemia six times a day consecutively for one month didn’t reduce BP, but endothelium-dependent vasodilation was shown to improve in healthy subjects [24]. The study of Banks *et al*. showed that nine consecutive days of once-daily LRIC treatment including four cycles of 5min ischemia/5min reperfusion cannot lead to a drop in BP [20]. Jones *et al.* also demonstrated that LRIC treatment which was three times a week for eight weeks improved endothelial function without lowering BP [18]. However, another study by Jones *et al.* showed a significant decline in BP after a 7-day intervention of once-daily LRIC therapy [12]. In this study, participants were recruited with an average baseline BP of 138±6/73±6 mmHg which should be defined as prehypertension rather than healthy volunteers. In summary, LRIC seems to have no significant effect on BP in normotensive or healthy volunteers (floor effect)(See Table 1).

**Table 2:** The effect of LRIC on BP in patients with CVD or hypertension.

Study	No. Participants	Design	Comparator	LRIC protocol	Frequency, Duration	Antihypertensive effect
Kuusik [33]	111, PAD	RCT	Sham control	4×5min ischemia/5min reperfusion	Once-only	NO
He [23]	49, AIS	RCT	Sham control	4×5min ischemia/5min reperfusion	Twice-only	NO
Kepler [32]	98, vascular surgery	RCT	Sham control	4×5min ischemia/5min reperfusion	Once-only	NO
Zhao [31]	20, AIS	Cohort	None	3×5min ischemia/5min reperfusion	Once-only	NO
England [30]	26, AIS	RCT	Sham control	4×5min ischemia/5min reperfusion	Once-only	NO
Li [26]	10, MCA stenosis	Cohort	None	5×5min ischemia/5min reperfusion	Once-only	NO
Meng [28]	58, SIAS	RCT	Sham control	5×5min ischemia/5min reperfusion	2×daily, 30 days	NO
Zagidulin [25]	30, angina pectoris	RCT	Sham control	3×5min ischemia/5min reperfusion	Once-only	YES
Medias [11]	1, normo-/pre-HTN	Case study	Self	3×5min ischemia /5min reperfusion	2×daily, 3 days	YES
Jones [12]	13, pre-HNT	Cohort	None	4×5min ischemia/5min reperfusion	1×daily, 7 days	YES
Medias [13]	1, normo-/pre-HTN	Case study	Self	3×5min ischemia	2×daily, 7 days	YES
Medias [14]	1, normo-/pre-HTN	Case study	Self	3×5min ischemia	2×daily, 10 days	YES
Medias [19]	1, normo-/pre-HTN	Case study	Self	3×5min ischemia	1×daily, 10 days	NO
Pujara [29]	20, CF LVADs	RCT	Control	3×5min ischemia/5min reperfusion	2×daily, 30 days	YES
Pryds [15]	22, CIHF	Cohort	None	4×5min ischemia/5min reperfusion	1×daily, 1 month	YES
Tong [16]	15, HTN	Cohort	None	3×5min ischemia/5min reperfusion	1×daily, 1 month	YES

The cuff pressure is about 200-220mmHg or at 20/50mmHg above systolic BP to induce ischemia. PAD, peripheral arterial disease; AIS, acute ischemic stroke; MCA, middle cerebral artery; SIAS, symptomatic intracranial arterial stenosis; HTN, hypertension, CF LVADs, continuous flow left ventricular assist devices; CIHF, chronic ischemic heart failure.

### 2.2 The effect of LRIC on BP in patients with CVD or hypertension

#### 2.2.1 The effect of once-only LRIC on BP

By evaluating the safety and feasibility of LRIC in patients with unilateral middle cerebral artery (MCA) stenosis, Li *et al.* reported that once-only LRIC protocol did not affect BP and heart rate in these patients [26]. The study of England *et al.* showed that once-only LRIC therapy had neither short-term nor delayed effect on central arterial pressure in patients with acute ischemic stroke (AIS) [30]. Other studies also draw similar conclusions that once or twice LRIC treatment only was not enough to reduce BP in patients with CVD [23, 31-33]. Meng *et al.* compared changes in BP before and after each LRIC treatment in patients with symptomatic intracranial artery stenosis (SIAS) and found that LRIC had no short-term hypotensive effect. However, BP levels before and after one month of therapy were not measured in this study, thus, the effect of long-term repeated LRIC on BP cannot be noticed from the result of this research [28]. Zagidullin’s study of 30 angina pectoris patients was the only one reporting that once-only LRIC training could lead to a drop in peripheral systolic BP [25]. Therefore, we conclude that once-only LRIC therapy seems not enough to reduce BP in individuals with CVD or hypertension (See Table 2).

#### 2.2.2 The effect of long-term repeated LRIC on BP

Medias is the first scholar who explored whether LRIC can reduce BP. He carried out a series of self-control studies by implementing LRIC on himself, a normotensive or prehypertensive person, and found that twice-daily LRIC treatment had both a short-term and delayed BP-lowering effect [11, 13, 14]. However, this effect disappeared in the circumstance of once-daily LRIC therapy [19]. Pujara *et al.* found that twice-daily LRIC performed over a period of one month could result in a significant reduction in average arterial pressure (MAP) in patients with continuous flow left ventricular assist devices (CF LVADs) [29]. Pyrds *et al.* reported that LRIC performed once a day for one month could reduce systolic BP in patients with chronic ischemic heart failure (CHIF)[15]. However, office BP measurement is not ideal compared with ambulatory BP measurement (ABPM). The study by Tong *et al.* demonstrated the impact of chronic LRIC on BP by using both office BP measurement and ABPM for the first time, which showed that one month of once-daily LRIC treatment could lead to a significant fall in systolic BP and diastolic BP in patients with hypertension [16]. In a word, long-term repeated LRIC therapy seems to have a potential antihypertensive effect in those with hypertension or CVD (See Table 2).

### 2.3 Speculation of long-term repeated LRIC may have an antihypertensive effect on hypertension

Considering all the available evidence mentioned above, the following speculations can be made. In summary of 8 clinical studies including 192 subjects, the first speculation is that neither LRIC performed once nor repeated for a long time has a significant BP-lowering effect on healthy volunteers with normal BP [18, 20-22, 24-27]. Secondly, after analyzing 8 studies involving 402 patients with CVD or hypertension, we confer that LRIC treatment performed only once seems insufficient to trigger the BP-lowering effect [23, 25, 26, 28, 30-33]. In addition, we presume that long-term repeated LRIC therapy may provide an alternative applicable intervention to treat hypertension, evidence of which was from 71 subjects involved in 8 studies [11-17, 19, 29].

Our team conducted an animal experiment and designed a clinical trial to further verify these speculations. Our completed animal experiment demonstrated that LRIC therapy once daily for six consecutive weeks could reduce MAP and attenuate vascular remolding by regulating immune or inflammatory responses in spontaneously hypertensive rats (SHR), but these changes did not occur in normotensive Wistar-Kyoto rats (WKY) [34]. A clinical trial was designed by us to demonstrate the antihypertensive effect of chronic LRIC is recruiting participants with prehypertension and mild hypertension currently (NCT03566654).

In a word, long-term repeated LRIC seems to be a promising novel antihypertensive therapy for hypertension. However, the mechanisms of the BP-lowering effect by chronic LRIC therapy still need to be clarified. Next, we will explore the intersections between the pathogenesis of hypertension and the endogenous protection mechanisms of LRIC to clarify this.

## 3. Potential mechanisms of the antihypertensive effect of LRIC

**Figure 1.**
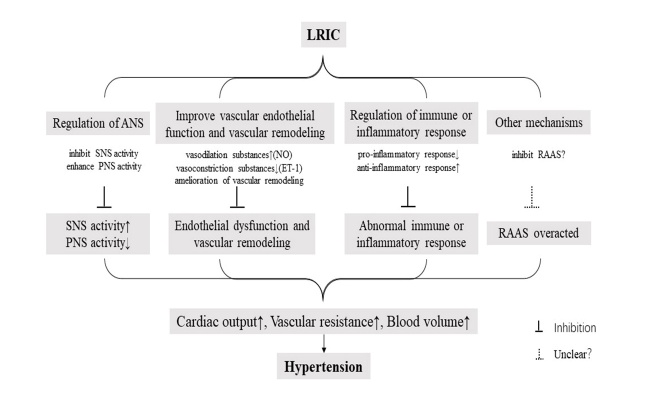
Potential mechanisms of the antihypertensive effect of LRIC.

BP homeostasis is determined by the complex interplay of cardiac output, peripheral vascular resistance, and blood volume. Any abnormality in the process of BP regulation can directly or indirectly lead to sustained elevated BP. Activated SNS and RAAS, chronic immune or inflammation response, and aberrant vascular endothelial function are well-established pathogenesis of hypertension [2]. Existing studies have presented many intersections between the endogenous protective mechanisms of LRIC and the pathogenesis of hypertension [5-7]. Therefore, it is reasonable to hypothesize that LRIC can repress the pathogenesis of hypertension through multiple pathways, thereby exerting a BP-lowering effect (Fig. 1).

### 3.1 Regulation of autonomic nervous system

Patients with hypertension are usually accompanied by continuous imbalance of the autonomic nervous system (ANS), this imbalance is characterized by increased SNS activity and decreased parasympathetic nerve system (PNS) activity [35]. Current treatments aiming to inhibit SNS activity, such as central SNS suppressing drugs, peripheral alpha- and beta-adrenergic receptor blockers, and renal sympathetic denervation, can significantly reduce BP[36]. Aside from SNS inhibition, stimulation of the PNS pathway can reduce BP as well [37, 38]. Therefore, restoring the balance of ANS is one of the important targets for antihypertensive therapy. LRIC treatment has been demonstrated by preclinical and clinical studies to have a positive effect on ANS.

#### 3.1.1 Evidence from animal experiments

In animal experiments, Ogawa *et al*. found that sympathetic nerves partly mediated the protective effect of ischemic conditioning against injuries in rats caused by renal ischemia-reperfusion, which could be offset by renal denervation [39]. Tsutsui *et al.* demonstrated that ischemic conditioning played protective roles in the rat model of ischemia/reperfusion-induced acute kidney injury for both the ischemia period and the reperfusion period. It suppressed the enhanced renal SNS activity during ischemia and decreased norepinephrine release during reperfusion time [40]. Apart from the sympathoinhibitory effect, LRIC played an important role in enhancing PNS activity as well. Through constructing multiple myocardial ischemia-reperfusion injury models, some others found that vagotomy, inhibition of the dorsal motor nucleus of the vagus (DMV), or atropine administration could eliminate the protective effects of LRIC [41-45]. Furthermore, stimulation of the vagus or DMV neurons could mimic the protective effect of LRIC [46-48].

#### 3.1.2 Evidence from clinical studies

In clinical studies, Lambert *et al.* reported that LRIC could attenuate and delay SNS activation induced by ischemia-reperfusion. In his study of 30 healthy volunteers, the randomized intervention of LRIC or no-LRIC was applied right before experimental ischemia-reperfusion. Participants in the control group had a 70% increase of SNS activity in the early phase of ischemia and a 101% increase in the late phase; those in the LRIC group only had a 40% increase in SNS activity which was delayed to the late phase of ischemia [49]. The study by Enko *et al*. showed that LRIC could enhance the activity of PNS and induce vasodilation in healthy volunteers [50]. In summary, we suppose that LRIC can lead to a drop in BP by decreasing SNS activity and increasing PNS activity.

### 3.2 Improve vascular endothelial function and vascular remodeling

The endothelium is a key regulator of vascular tone, which can release a host of vasoactive substances including vasodilating factors and vasoconstricting factors to regulate vascular resistance and influence BP [2, 51, 52]. Patients with hypertension are usually accompanied by endothelial dysfunction which is characterized by decreasing the production or bioavailability of endothelium-derived relaxing factors, especially nitric oxide (NO), and increasing the release or responsiveness of vasoconstrictor factors [2, 51, 52]. Some studies supposed that endothelial dysfunction preceded the establishment of essential hypertension, but others suggested that endothelial dysfunction was a consequence of elevated BP. It seems to be a vicious cycle as deterioration of one may worsen the other, promoting pathological vascular remodeling and leading to poor prognosis [51-53]. Hence the early recognition and timely management of endothelial dysfunction and vascular remodeling are beneficial to reduce BP and prevent hypertension associated end-organ damage [54].

#### 3.2.1 Evidence from animal experiments

Animal experiments have suggested that endothelium-derived factors play an important role in the protective effect of RIC. Rassaf *et al.* found that LRIC therapy increased the level of circulating nitrite (a key reservoir for NO) in mice, which was associated with the cardioprotection of LRIC[55]. He *et al.* reported that up-regulation of PI3K/Akt/eNOS/NO pathway mediated the protective role of RIC in a rat model of liver transplantation [56]. Aggarwal *et al.* reviewed the role of endothelium in RIC-induced cardioprotection, especially underlining endothelium-derived factors. He concluded that NO was a central mediator in the protective effect of RIC[57]. Other vasoactive substances, such as adenosine, prostacyclin, calcitonin gene-related peptides, angiotensin II (Ang II), and thromboxane A2, may play roles as well [57-61]. Besides, Gao *et al.* demonstrated that chronic LRIC benefited both the conducting and the resistance arteries of SHR. It ameliorated hypertrophic vascular remodeling by suppressing the deposition of the extracellular matrix in conducting arteries and decreasing the collagen production and the degree of smooth muscle cell hypertrophy in the resistance arteries [34].

#### 3.2.2 Evidence from clinical studies

The fact that the improvement of endothelial function and vascular remodeling by RIC procedure has been observed in numerous clinical studies. Kimura *et al.* [24] and *Jones et al.* [12, 18] found that repeated LRIC treatment augmented endothelium-dependent vasodilation in healthy adults. Moro *et al.* reported that once-only LRIC therapy was sufficient to improve endothelium-dependent vasodilation in healthy or hypertensive elderly subjects [62]. In stroke patients, the evidence that endothelium-dependent vasodilation improved after two weeks of LRIC treatment has been demonstrated by Hyngstrom *et al.* [63]. Similarly, Liang *et al.* reported that chronic LRIC improved endothelium-dependent vasodilation in coronary heart disease (CHD) patients [64]. Apart from improving vasodilation, LRIC may alleviate continuous contraction of the blood vessel by inhibiting the synthesis of endothelin-1 (ET-1) [65-67]. Gao *et al.* demonstrated that chronic LRIC could improve the situation of vascular stiffness in patients with prehypertension and mild hypertension, which was consistent with the results in her animal experiment [34]. In conclusion, we hypothesize that LRIC may exert its antihypertensive effect by improving endothelial function and vascular remodeling.

### 3.3 Regulation of immune/inflammatory responses

Numerous preclinical and clinical studies highlight the relationship between hypertension and prolonged activation of immune or inflammatory responses [2]. Chronic activation of innate and adaptive immune systems promotes the accumulation of immune cells and autoantibodies in the heart, brain, kidney, and blood vessel, which leads to hypertension by weakening BP regulation of these organs [2, 68, 69]. Researches have shown that immune-targeting therapies not only reduced BP in experimental hypertensive animals [70, 71] but also improved BP control in hypertensive patients with autoimmune diseases [72, 73]. Although the risk-benefit ratio of immuno-targeting therapies seems to be high, it is still being considered as one of the important approaches in treating hypertension and CVD in the future [69, 74]. In contrast, LRIC could exert its protective effect by regulating immune/inflammatory response with few side effects [75], which might be a therapy to hypertension of good potential.

#### 3.3.1 Evidence from animal experiments

In a lipopolysaccharide-induced mouse model, Kim *et al.* found that LRIC could attenuate inflammatory response and improve the survival rate of mice. The mechanisms of this intervention were that LRIC decreased circulating pro-inflammatory (such as TNF-a, IL-1β, and IL-6), increased plasma anti-inflammatory factors (such as IL-10), and reduced infiltration of neutrophil in the liver [76]. Zhou *et al.* reported that LRIC improved lung function of a cardiopulmonary bypass (CPB) induced pulmonary injury model in rats through increasing IL-10 and IL-4 anti-inflammatory cytokines and reducing neutrophil infiltration in alveoli [77]. Wei *et al.* proved that chronic LRIC could reduce the infarct size and improve survival outcome in a myocardial infarction model of rats. Which was mediated by lowering the production of circulating TNF-α, IL-1β, and MCP-1 and lessening the macrophage/neutrophil infiltration in the infarct border zone [78]. Chen *et al.* [79] and Heusch *et al.* [80] regarded that LRIC could shift splenic immune response toward a favorable systemic immune milieu, thereby, protecting the brain and heart from ischemic injury. Gao *et al.* demonstrated that LRIC had a direct immunomodulation effect on SHR. In her study, once-daily LRIC for six consecutive weeks not only decreased the inflammatory profiles including circulating monocyte, natural killer T cells, and pro-inflammatory factors (TNF-a, IL-1β, and CXCL1) but also increased the anti-inflammatory factors (IL-10 and IL-13) [34].

#### 3.3.2 Evidence from clinical studies

Contrary to animal experiments, most clinical trials were unable to confirm the effect of RIC on inflammatory response. Konstantinov *et al.* found that LRIC could suppress the transcription of proinflammatory genes in human leukocytes [81]. Shimizu *et al.* demonstrated these changes in proinflammatory gene expression could lead to functional changes in neutrophils, including reduced adhesion, phagocytosis, and exocytosis [82]. He *et al.* carried out an RCT in 90 elderly patients following colon surgery and found that LRIC could improve postoperative cognitive function by inhibiting inflammatory response [83]. RCT conducted by Wang *et al.* showed that LRIC may be a protective therapy in coronary artery bypass graft surgery, which can be partially attributed to the reduced production of inflammatory factors(IL-6, IL-8, TNF-α) [84]. In summary, we speculate that LRIC may reduce BP by modulating immune/inflammatory response.

### 3.4 Other mechanisms

Overactivated RAAS plays an important part in the pathogenesis of hypertension. Most current drugs for hypertension, such as angiotensin-converting enzyme inhibitors, angiotensin receptor blocks, and mineralocorticoid antagonists, aim to block the influence of RAAS on BP [36]. However, only a few studies reported the impact of LRIC on RAAS. One clinical study showed that LRIC might exert its neuroprotective effects by reducing the level of circulating Ang II in stroke patients [65]. However, an animal experiment showed that LRIC might resist myocardial ischemia-reperfusion injury through the preconditioning of Ang II [85]. Therefore, it remains to be further explored whether LRIC reduces BP by inhibiting RAAS.

The increased releasing of stromal derived factor 1α (SDF-1α) may be another potential mechanism. The study from Tong *et al.* has demonstrated that plasma SDF-1α increased after chronic LRIC therapy. This increase of SDF-1α was associated with a fall in BP and an improvement in endothelial function [16]. Evidence from another study also demonstrated that LRIC might exert its protective effects by increasing SDF-1α [86].

## 4. Future Perspectives of LRIC in the treatment of hypertension

It seems that chronic LRIC has a potential antihypertensive effect on hypertension from the available data so far. However, further studies are required to clarify this phenomenon and the exact mechanisms involved. The ongoing HOPE study (NCT03566654) aims to explore the BP-lowering effect of chronic LRIC on patients with prehypertension or early-stage hypertension. The SERIC-EH study (NCT03945305) intends to evaluate the safety and efficacy of chronic LRIC on hypertensive patients who are taking antihypertensive drugs and still lack normal BP control. Other ongoing studies are accessing the BP-lowering effect of LRIC on women with pre-eclampsia (NCT03323762), patients with chronic kidney disease and hypertension (NCT03236350), and healthy volunteers or essential hypertensive patients (NCT02414997). These studies will provide compelling evidence for the safety and efficacy of LRIC on the treatment of hypertension in a different population, thereby providing a novel and effective alternative treatment.

However, we are still facing several problems about implementing chronic LRIC on more hypertensive patients. Firstly, the antihypertensive effects of chronic LRIC should be further validated in large-scale and rigorous RCTs in patients with elevated BP. Secondly, despite the beneficial effects of LRIC on multiple organs have been demonstrated, the optimal scheme of LRIC for efficiently reducing BP remains to be explored. Thirdly, the mechanisms of LRIC are mostly investigated in the ischemia-reperfusion injury model of different organs, which seems cannot be directly applied in the case of hypertension. What’s more, the impact of co-morbidities and co-medications on RIC therapy in hypertension patients must be considered in future clinical trials based on previous experience and evidence [87, 88]. Most importantly, accurate BP measurement (using ABPM as a gold standard) should be adopted with serious-minded across different studies. Therefore, future efforts should be focused on addressing these questions mentioned above.

## 5. Conclusion

In conclusion, chronic LRIC may restrain the pathogenesis of hypertension and lower BP through multiple pathways including regulation of ANS, improvement of vascular endothelial function, and modulation of immune/inflammatory responses. However, before widely applying chronic LRIC as a treatment of hypertension, further studies are warranted to build a solid foundation so as to validate the beneficial effect of LRIC and its adverse effects.

## References

[1] GBD 2019 Risk Factors Collaborators (2020). Global burden of 87 risk factors in 204 countries and territories, 1990-2019: a systematic analysis for the Global Burden of Disease Study 2019. Lancet, 396:1223-1249. 10.1016/S0140-6736(20)30752-2 PMC7566194 33069327

[2] Oparil S, Acelajado MC, Bakris GL, Berlowitz DR, Cifkova R, Dominiczak AF, et al. (2018). Hypertension. Nat Rev Dis Primers, 4:18014. 29565029 10.1038/nrdp.2018.14 PMC6477925

[3] Beaney T, Schutte AE, Stergiou GS, Borghi C, Burger D, Charchar F, et al. (2020). May Measurement Month 2019: The Global Blood Pressure Screening Campaign of the International Society of Hypertension. Hypertension, 76:333-341. 32419505 10.1161/HYPERTENSIONAHA.120.14874

[4] Wang Z, Chen Z, Zhang L, Wang X, Hao G, Zhang Z, et al. (2018). Status of Hypertension in China: Results From the China Hypertension Survey, 2012-2015. Circulation, 137:2344-2356. 29449338 10.1161/CIRCULATIONAHA.117.032380

[5] Heusch G, Bøtker HE, Przyklenk K, Redington A, Yellon D (2015). Remote ischemic conditioning. J Am Coll Cardiol, 65:177-195. 25593060 10.1016/j.jacc.2014.10.031 PMC4297315

[6] Kleinbongard P, Skyschally A, Heusch G (2017). Cardioprotection by remote ischemic conditioning and its signal transduction. Pflugers Arch, 469:159-181. 27928644 10.1007/s00424-016-1922-6

[7] Hess DC, Blauenfeldt RA, Andersen G, Hougaard KD, Hoda MN, Ding Y, et al. (2015). Remote ischaemic conditioning-a new paradigm of self-protection in the brain. Nat Rev Neurol, 11:698-710. 26585977 10.1038/nrneurol.2015.223

[8] Pickard JM, Bøtker HE, Crimi G, Davidson B, Davidson SM, Dutka D, et al. (2015). Remote ischemic conditioning: from experimental observation to clinical application: report from the 8th Biennial Hatter Cardiovascular Institute Workshop. Basic Res Cardiol, 110:453. 25449895 10.1007/s00395-014-0453-6 PMC4250562

[9] Heusch G, Rassaf T (2016). Time to Give Up on Cardioprotection? A Critical Appraisal of Clinical Studies on Ischemic Pre-, Post-, and Remote Conditioning. Circ Res, 119:676-695. 27539973 10.1161/CIRCRESAHA.116.308736

[10] Heusch G (2020). Myocardial ischaemia-reperfusion injury and cardioprotection in perspective. Nat Rev Cardiol, 17:773-789. 32620851 10.1038/s41569-020-0403-y

[11] Madias JE (2011). Effect of serial arm ischemic preconditioning sessions on the systemic blood pressure of a normotensive subject. Med Hypotheses, 76:503-506. 21194848 10.1016/j.mehy.2010.12.002

[12] Jones H, Hopkins N, Bailey TG, Green DJ, Cable NT, Thijssen DH (2014). Seven-day remote ischemic preconditioning improves local and systemic endothelial function and microcirculation in healthy humans. Am J Hypertens, 27:918-925. 24627443 10.1093/ajh/hpu004

[13] Madias JE, Koulouridis I (2014). Effect of repeat twice daily sessions of remote ischemic conditioning over the course of one week on blood pressure of a normotensive/prehypertensive subject. Int J Cardiol, 176:1076-1077. 25127977 10.1016/j.ijcard.2014.07.132

[14] Madias JE (2015). Sustained blood pressure lowering effect of twice daily remote ischemic conditioning sessions in a normotensive/prehypertensive subject. Int J Cardiol, 182:392-394. 25596469 10.1016/j.ijcard.2014.12.159

[15] Pryds K, Nielsen RR, Jorsal A, Hansen MS, Ringgaard S, Refsgaard J, et al. (2017). Effect of long-term remote ischemic conditioning in patients with chronic ischemic heart failure. Basic Res Cardiol, 112. 10.1007/s00395-017-0658-6 29071437

[16] Tong XZ, Cui WF, Li Y, Su C, Shao YJ, Liang JW, et al. (2019). Chronic remote ischemic preconditioning-induced increase of circulating hSDF-1α level and its relation with reduction of blood pressure and protection endothelial function in hypertension. J Hum Hypertens, 33:856-862. 30631131 10.1038/s41371-018-0151-1

[17] Epps J, Dieberg G, Smart NA (2016). Repeat remote ischaemic pre-conditioning for improved cardiovascular function in humans: A systematic review. IJC Heart and Vasculature, 11:55-58. 28616526 10.1016/j.ijcha.2016.03.003 PMC5441349

[18] Jones H, Nyakayiru J, Bailey TG, Green DJ, Cable NT, Sprung VS, et al. (2015). Impact of eight weeks of repeated ischaemic preconditioning on brachial artery and cutaneous microcirculatory function in healthy males. Eur J Prev Cardiol, 22:1083-1087. 25147345 10.1177/2047487314547657

[19] Madias JE (2015). Absence of a sustained blood pressure lowering effect of once daily remote ischemic conditioning sessions in a normotensive/ prehypertensive subject. Int J Cardiol, 184:307-309. 25731845 10.1016/j.ijcard.2015.02.089

[20] Banks L, Wells GD, Clarizia NA, Jean-St-Michel E, McKillop AL, Redington AN, et al. (2016). Short-term remote ischemic preconditioning is not associated with improved blood pressure and exercise capacity in young adults. Appl physiol nutr metab, 41:903-906. 27439445 10.1139/apnm-2016-0024

[21] Khaliulin I, Fleishman AN, Shumeiko NI, Korablina TV, Petrovskiy SA, Ascione R, et al. (2019). Neuro-autonomic changes induced by remote ischemic preconditioning (RIPC) in healthy young adults: Implications for stress. Neurobiol Stress, 11. 10.1016/j.ynstr.2019.100189 PMC6675953 31388518

[22] Mueller J, Taebling M, Oberhoffer R (2019). Remote Ischemic Preconditioning Has No Short Term Effect on Blood Pressure, Heart Rate, and Arterial Stiffness in Healthy Young Adults. Front Physiol, 10. 10.3389/fphys.2019.01094 PMC6712092 31496958

[23] He YD, Guo ZN, Qin C, Jin H, Zhang P, Abuduxukuer R, et al. (2020). Remote ischemic conditioning combined with intravenous thrombolysis for acute ischemic stroke. Ann Clin Transl Neurol, 7:972-979. 32472628 10.1002/acn3.51063 PMC7318096

[24] Kimura M, Ueda K, Goto C, Jitsuiki D, Nishioka K, Umemura T, et al. (2007). Repetition of Ischemic Preconditioning Augments Endothelium-Dependent Vasodilation in Humans. Arterioscler Thromb Vasc Biol 27:1403-1410. 17446439 10.1161/ATVBAHA.107.143578

[25] Zagidullin N, Scherbakova E, Safina Y, Zulkarneev R, Zagidullin S (2016). The impact of remote ischemic preconditioning on arterial stiffness and heart rate variability in patients with angina pectoris. J Clin Med, 5(7):60. 10.3390/jcm5070060 PMC4961991 27348009

[26] Li S, Ma C, Shao G, Esmail F, Hua Y, Jia L, et al. (2015). Safety and Feasibility of Remote Limb Ischemic Preconditioning in Patients With Unilateral Middle Cerebral Artery Stenosis and Healthy Volunteers. Cell Transplant, 24:1901-1911. 25198862 10.3727/096368914X683520

[27] Grau M, Kollikowski A, Bloch W (2016). Remote ischemia preconditioning increases red blood cell deformability through red blood cell-nitric oxide synthase activation. Clin Hemorheol Microcirc, 63:185-197. 26890111 10.3233/CH-152039

[28] Meng R, Ding Y, Asmaro K, Brogan D, Meng L, Sui M, et al. (2015). Ischemic Conditioning Is Safe and Effective for Octo- and Nonagenarians in Stroke Prevention and Treatment. Neurotherapeutics, 12:667-677. 25956401 10.1007/s13311-015-0358-6 PMC4489956

[29] Pujara D, Mallidi HR, Cohn WE, Anand J, Frazier OH, Singh SK (2016). Investigating a novel synergy applying remote ischemic conditioning to modulate the altered physiology of continuous flow left ventricular assist devices, to reduce stroke and other adverse effects: The impulse trial pilot results. J Heart Lung Transplant, 35:S393-S394.

[30] England TJ, Hedstrom A, O'Sullivan S, Donnelly R, Barrett DA, Sarmad S, et al. (2017). RECAST (Remote Ischemic Conditioning after Stroke Trial): A Pilot Randomized Placebo Controlled Phase II Trial in Acute Ischemic Stroke. Stroke, 48:1412-1415. 28265014 10.1161/STROKEAHA.116.016429

[31] Zhao W, Che R, Li S, Ren C, Li C, Wu C, et al. (2018). Remote ischemic conditioning for acute stroke patients treated with thrombectomy. Ann Clin Transl Neurol, 5:850-856. 30009202 10.1002/acn3.588 PMC6043766

[32] Kepler T, Kuusik K, Lepner U, Starkopf J, Zilmer M, Eha J, et al. (2019). The Effect of Remote Ischaemic Preconditioning on Arterial Stiffness in Patients Undergoing Vascular Surgery: A Randomised Clinical Trial. Eur J Vasc Endovasc Surg, 57:868-875. 31126835 10.1016/j.ejvs.2018.12.002

[33] Kuusik K, Kepler T, Zilmer M, Eha J, Vahi M, Kals J (2019). Effects of Remote Ischaemic Preconditioning on Arterial Stiffness in Patients Undergoing Lower Limb Angiographic Procedures: A Randomised Clinical Trial. Eur J Vasc Endovasc Surg, 58:875-882. 31648881 10.1016/j.ejvs.2019.06.004

[34] Gao Y, Ren C, Li X, Yu W, Li S, Li H, et al. (2021). Ischemic Conditioning Ameliorated Hypertension and Vascular Remodeling of Spontaneously Hypertensive Rat via Inflammatory Regulation. Aging Dis, 12:116-131. 33532132 10.14336/AD.2020.0320 PMC7801289

[35] Mancia G, Grassi G (2014). The autonomic nervous system and hypertension. Circ Res, 114:1804-1814. 24855203 10.1161/CIRCRESAHA.114.302524

[36] Whelton PK, Carey RM, Aronow WS, Casey DE Jr, Collins KJ, Dennison Himmelfarb C, et al. (2018). 2017 ACC/AHA/AAPA/ABC/ACPM/AGS/APhA/ ASH/ASPC/NMA/PCNA Guideline for the Prevention, Detection, Evaluation, and Management of High Blood Pressure in Adults: Executive Summary: A Report of the American College of Cardiology/American Heart Association Task Force on Clinical Practice Guidelines. Hypertension, 71:1269-1324. 29133354 10.1161/HYP.0000000000000066

[37] Moreira TS, Antunes VR, Falquetto B, Marina N (2018). Long-term stimulation of cardiac vagal preganglionic neurons reduces blood pressure in the spontaneously hypertensive rat. J Hypertens, 36:2444-2452. 30045362 10.1097/HJH.0000000000001871

[38] Wustmann K, Kucera JP, Scheffers I, Mohaupt M, Kroon AA, de Leeuw PW, et al. (2009). Effects of chronic baroreceptor stimulation on the autonomic cardiovascular regulation in patients with drug-resistant arterial hypertension. Hypertension, 54:530-536. 19620513 10.1161/HYPERTENSIONAHA.109.134023

[39] Ogawa T, Mimura Y, Kaminishi M (2002). Renal denervation abolishes the protective effects of ischaemic preconditioning on function and haemodynamics in ischaemia-reperfused rat kidneys. Acta Physiol Scand, 174:291-297. 11906329 10.1046/j.1365-201x.2002.00944.x

[40] Tsutsui H, Tanaka R, Yamagata M, Yukimura T, Ohkita M, Matsumura Y (2013). Protective effect of ischemic preconditioning on ischemia/reperfusion-induced acute kidney injury through sympathetic nervous system in rats. Eur J Pharmacol, 718:206-212. 24036256 10.1016/j.ejphar.2013.08.032

[41] Basalay M, Barsukevich V, Mastitskaya S, Mrochek A, Pernow J, Sjöquist PO, et al. (2012). Remote ischaemic pre- and delayed postconditioning - similar degree of cardioprotection but distinct mechanisms. Exp Physiol, 97:908-917. 22427438 10.1113/expphysiol.2012.064923 PMC3470925

[42] Basalay M, Barsukevich V, Mrochek A, Gourine AV, Gourine A.2013. Right vs left vagus and cardioprotection conferred by remote ischaemic pre- and perconditioning. Eur Heart J, 34: 3697.

[43] Donato M, Buchholz B, Rodríguez M, Pérez V, Inserte J, García-Dorado D, et al. (2013). Role of the parasympathetic nervous system in cardioprotection by remote hindlimb ischaemic preconditioning. Exp Physiol, 98:425-434. 22872660 10.1113/expphysiol.2012.066217

[44] Mastitskaya S, Marina N, Gourine A, Gilbey MP, Spyer KM, Teschemacher AG, et al. (2012). Cardioprotection evoked by remote ischaemic preconditioning is critically dependent on the activity of vagal pre-ganglionic neurones. Cardiovasc Res, 95:487-494. 22739118 10.1093/cvr/cvs212 PMC3422080

[45] Lieder HR, Kleinbongard P, Skyschally A, Hagelschuer H, Chilian WM, Heusch G (2018). Vago-Splenic Axis in Signal Transduction of Remote Ischemic Preconditioning in Pigs and Rats. Circ Res, 123:1152-1163. 30359199 10.1161/CIRCRESAHA.118.313859 PMC7304918

[46] Buchholz B, Kelly J, Muñoz M, Bernatené EA, Méndez Diodati N, González Maglio DH, et al. (2018). Vagal stimulation mimics preconditioning and postconditioning of ischemic myocardium in mice by activating different protection mechanisms. Am J Physiol Heart Circ Physiol, 314:H1289-h1297. 29631370 10.1152/ajpheart.00286.2017

[47] Uitterdijk A, Yetgin T, te Lintel Hekkert M, Sneep S, Krabbendam-Peters I, van Beusekom HM, et al. (2015). Vagal nerve stimulation started just prior to reperfusion limits infarct size and no-reflow. Basic Res Cardiol, 110:508. 26306761 10.1007/s00395-015-0508-3 PMC4549380

[48] Heusch G (2017). Vagal Cardioprotection in Reperfused Acute Myocardial Infarction. JACC Cardiovasc Interv, 10:1521-1522. 28797428 10.1016/j.jcin.2017.05.063

[49] Lambert EA, Thomas CJ, Hemmes R, Eikelis N, Pathak A, Schlaich MP, et al. (2016). Sympathetic nervous response to ischemia-reperfusion injury in humans is altered with remote ischemic preconditioning. Am J Physiol Heart Circ Physiol, 311:H364-370. 27288436 10.1152/ajpheart.00369.2016

[50] Enko K, Nakamura K, Yunoki K, Miyoshi T, Akagi S, Yoshida M, et al. (2011). Intermittent arm ischemia induces vasodilatation of the contralateral upper limb. J Physiol Sci, 61:507-513. 21901641 10.1007/s12576-011-0172-9 PMC10718035

[51] Konukoglu D, Uzun H (2017). Endothelial Dysfunction and Hypertension. Adv Exp Med Biol, 956:511-540. 28035582 10.1007/5584_2016_90

[52] Mordi I, Mordi N, Delles C, Tzemos N (2016). Endothelial dysfunction in human essential hypertension. J Hypertens, 34:1464-1472. 27203578 10.1097/HJH.0000000000000965

[53] Laurent S, Boutouyrie P (2015). The structural factor of hypertension: large and small artery alterations. Circ Res, 116:1007-1021. 25767286 10.1161/CIRCRESAHA.116.303596

[54] Modena MG, Bonetti L, Coppi F, Bursi F, Rossi R (2002). Prognostic role of reversible endothelial dysfunction in hypertensive postmenopausal women. J Am Coll Cardiol, 40:505-510. 12142118 10.1016/s0735-1097(02)01976-9

[55] Rassaf T, Totzeck M, Hendgen-Cotta UB, Shiva S, Heusch G, Kelm M (2014). Circulating nitrite contributes to cardioprotection by remote ischemic preconditioning. Circ Res, 114:1601-1610. 24643960 10.1161/CIRCRESAHA.114.303822

[56] He N, Jia JJ, Li JH, Zhou YF, Lin BY, Peng YF, et al. (2017). Remote ischemic perconditioning prevents liver transplantation-induced ischemia/reperfusion injury in rats: Role of ROS/RNS and eNOS. World J Gastroenterol, 23:830-841. 28223727 10.3748/wjg.v23.i5.830 PMC5296199

[57] Aggarwal S, Randhawa PK, Singh N, Jaggi AS (2016). Preconditioning at a distance: Involvement of endothelial vasoactive substances in cardioprotection against ischemia-reperfusion injury. Life Sci, 151:250-258. 26979771 10.1016/j.lfs.2016.03.021

[58] Steensrud T, Li J, Dai X, Manlhiot C, Kharbanda RK, Tropak M, et al. (2010). Pretreatment with the nitric oxide donor SNAP or nerve transection blocks humoral preconditioning by remote limb ischemia or intra-arterial adenosine. Am J Physiol Heart Circ Physiol, 299:H1598-1603. 20802131 10.1152/ajpheart.00396.2010

[59] Nakamura M, Nakakimura K, Matsumoto M, Sakabe T (2002). Rapid tolerance to focal cerebral ischemia in rats is attenuated by adenosine A1 receptor antagonist. J Cereb Blood Flow Metab, 22:161-170. 11823714 10.1097/00004647-200202000-00004

[60] Xiao L, Lu R, Hu CP, Deng HW, Li YJ (2001). Delayed cardioprotection by intestinal preconditioning is mediated by calcitonin gene-related peptide. Eur J Pharmacol, 427:131-135. 11557265 10.1016/s0014-2999(01)01231-6

[61] Oxman T, Arad M, Klein R, Avazov N, Rabinowitz B (1997). Limb ischemia preconditions the heart against reperfusion tachyarrhythmia. Am J Physiol, 273:H1707-1712. 9362234 10.1152/ajpheart.1997.273.4.H1707

[62] Moro L, Pedone C, Mondi A, Nunziata E, Antonelli Incalzi R (2011). Effect of local and remote ischemic preconditioning on endothelial function in young people and healthy or hypertensive elderly people. Atherosclerosis, 219:750-752. 21945497 10.1016/j.atherosclerosis.2011.08.046

[63] Hyngstrom AS, Nguyen JN, Wright MT, Tarima SS, Schmit BD, Gutterman DD, et al. (2020). Two weeks of remote ischemic conditioning improves brachial artery flow mediated dilation in chronic stroke survivors. J Appl Physiol (1985), 129:1348-1354. 33090908 10.1152/japplphysiol.00398.2020 PMC7792845

[64] Liang Y, Li YP, He F, Liu XQ, Zhang JY (2015). Long-term, regular remote ischemic preconditioning improves endothelial function in patients with coronary heart disease. Braz J Med Biol Res, 48:568-576. 25923462 10.1590/1414-431X20144452 PMC4470317

[65] Liu J, Ma X, Ji X, Li Y, Ding X, Li Y, et al. (2018). Effects of remote ischemic preconditioning on plasma endothelin-1, angiotensin II, and NO levels in patients with acute ischemic stroke. Chinese Journal of Public Health Engineering, 17(06):919-921.

[66] Wang N, Wang GS, Yu HY, Mi L, Guo LJ, Gao W (2014). [Myocardial protection of remote ischemic postconditioning during primary percutaneous coronary intervention in patients with acute ST-segment elevation myocardial infarction]. Beijing Da Xue Xue Bao Yi Xue Ban, 46:838-843. 25512268

[67] Jin X, Wang L, Li L, Zhao X (2019). Protective effect of remote ischemic pre-conditioning on patients undergoing cardiac bypass valve replacement surgery: A randomized controlled trial. Exp Ther Med, 17:2099-2106. 30867697 10.3892/etm.2019.7192 PMC6396008

[68] Zhang RM, McNerney KP, Riek AE, Bernal-Mizrachi C (2021). Immunity and Hypertension. Acta Physiol (Oxf), 231:e13487. 32359222 10.1111/apha.13487 PMC7606441

[69] Drummond GR, Vinh A, Guzik TJ, Sobey CG (2019). Immune mechanisms of hypertension. Nat Rev Immunol, 19:517-532. 30992524 10.1038/s41577-019-0160-5

[70] Bravo Y, Quiroz Y, Ferrebuz A, Vaziri ND, Rodríguez-Iturbe B (2007). Mycophenolate mofetil administration reduces renal inflammation, oxidative stress, and arterial pressure in rats with lead-induced hypertension. Am J Physiol Renal Physiol, 293:F616-623. 17567935 10.1152/ajprenal.00507.2006

[71] Marvar PJ, Hendy EB, Cruise TD, Walas D, DeCicco D, Vadigepalli R, et al. (2016). Systemic leukotriene B(4) receptor antagonism lowers arterial blood pressure and improves autonomic function in the spontaneously hypertensive rat. J Physiol, 594:5975-5989. 27230966 10.1113/JP272065 PMC5063948

[72] Herrera J, Ferrebuz A, MacGregor EG, Rodriguez-Iturbe B (2006). Mycophenolate mofetil treatment improves hypertension in patients with psoriasis and rheumatoid arthritis. J Am Soc Nephrol, 17:S218-225. 17130265 10.1681/ASN.2006080918

[73] Tsurko VV, Parnes E, Krasnosel'skiĭ M (2002). Clinical efficacy of xefocam and its effect on arterial pressure and heart rhythm variability in patients with rheumatoid arthritis in combination with arterial hypertension. Ter Arkh, 74:63-66. 12087911

[74] Liberale L, Ministrini S, Carbone F, Camici GG, Montecucco F (2021). Cytokines as therapeutic targets for cardio- and cerebrovascular diseases. Basic Res Cardiol, 116:23. 33770265 10.1007/s00395-021-00863-x PMC7997823

[75] Pearce L, Davidson SM, Yellon DM (2021). Does remote ischaemic conditioning reduce inflammation? A focus on innate immunity and cytokine response. Basic Res Cardiol, 116:12. 33629195 10.1007/s00395-021-00852-0 PMC7904035

[76] Kim YH, Yoon DW, Kim JH, Lee JH, Lim CH (2014). Effect of remote ischemic post-conditioning on systemic inflammatory response and survival rate in lipopolysaccharide-induced systemic inflammation model. J Inflamm (Lond), 11:16. 24904237 10.1186/1476-9255-11-16 PMC4046032

[77] Zhou X, Jiang R, Dong Y, Wang L (2017). Remote ischemic preconditioning attenuates cardiopulmonary bypass-induced lung injury. PLoS One, 12:e0189501. 29232398 10.1371/journal.pone.0189501 PMC5726632

[78] Wei M, Xin P, Li S, Tao J, Li Y, Li J, et al. (2011). Repeated remote ischemic postconditioning protects against adverse left ventricular remodeling and improves survival in a rat model of myocardial infarction. Circ Res, 108:1220-1225. 21474817 10.1161/CIRCRESAHA.110.236190

[79] Chen C, Jiang W, Liu Z, Li F, Yang J, Zhao Y, et al. (2018). Splenic responses play an important role in remote ischemic preconditioning-mediated neuroprotection against stroke. J Neuroinflammation, 15:167. 29807548 10.1186/s12974-018-1190-9 PMC5972448

[80] Heusch G (2019). The Spleen in Myocardial Infarction. Circ Res, 124:26-28. 30605405 10.1161/CIRCRESAHA.118.314331

[81] Konstantinov IE, Arab S, Kharbanda RK, Li J, Cheung MM, Cherepanov V, et al. (2004). The remote ischemic preconditioning stimulus modifies inflammatory gene expression in humans. Physiol Genomics, 19:143-150. 15304621 10.1152/physiolgenomics.00046.2004

[82] Shimizu M, Saxena P, Konstantinov IE, Cherepanov V, Cheung MM, Wearden P, et al. (2010). Remote ischemic preconditioning decreases adhesion and selectively modifies functional responses of human neutrophils. J Surg Res, 158:155-161. 19540519 10.1016/j.jss.2008.08.010

[83] He Z, Xu N, Qi S (2017). Remote ischemic preconditioning improves the cognitive function of elderly patients following colon surgery: A randomized clinical trial. Medicine (Baltimore), 96:e6719. 28445286 10.1097/MD.0000000000006719 PMC5413251

[84] Wang H, Lyu Y, Liao Q, Jin L, Xu L, Hu Y, et al. (2019). Effects of Remote Ischemic Preconditioning in Patients Undergoing Off-Pump Coronary Artery Bypass Graft Surgery. Front Physiol, 10:495. 31110480 10.3389/fphys.2019.00495 PMC6501551

[85] Singh D, Chopra K (2004). Evidence of the role of angiotensin AT(1) receptors in remote renal preconditioning of myocardium. Methods Find Exp Clin Pharmacol, 26:117-122. 15071610 10.1358/mf.2004.26.2.800064

[86] Davidson SM, Selvaraj P, He D, Boi-Doku C, Yellon RL, Vicencio JM, et al. (2013). Remote ischaemic preconditioning involves signalling through the SDF-1α/CXCR4 signalling axis. Basic Res Cardiol, 108:377. 23917520 10.1007/s00395-013-0377-6

[87] Heusch G (2017). Critical Issues for the Translation of Cardioprotection. Circ Res, 120:1477-1486. 28450365 10.1161/CIRCRESAHA.117.310820

[88] Schulz R, Andreadou I, Hausenloy DJ, Ferdinandy P (2020). Risk factors, co-morbidities, and co-medications in cardioprotection: Importance for translation. Br J Pharmacol, 177:5249-5251. 33219717 10.1111/bph.15294 PMC7679999

